# iLead—a transformational leadership intervention to train healthcare managers’ implementation leadership

**DOI:** 10.1186/s13012-016-0475-6

**Published:** 2016-07-29

**Authors:** Anne Richter, Ulrica von Thiele Schwarz, Caroline Lornudd, Robert Lundmark, Rebecca Mosson, Henna Hasson

**Affiliations:** 1 Procome Research Group, Medical Management Centre, Department of Learning, Informatics, Management and Ethics, Karolinska Institutet, 171 77 Stockholm, Sweden; 2 Department of Psychology, Stockholm University, 106 91 Stockholm, Sweden; 3 Unit for Implementation, Center for Epidemiology and Community Medicine (CES), Stockholm County Council, 171 29 Stockholm, Sweden; 4 Leadership, Evaluation and Organizational Development Research Group, Department of Learning, Informatics, Management and Ethics, Medical Management Centre, Karolinska Institutet, 171 77 Stockholm, Sweden

**Keywords:** Leadership training, Full range leadership model, Intervention, Change management

## Abstract

**Background:**

Leadership is a key feature in implementation efforts, which is highlighted in most implementation frameworks. However, in studying leadership and implementation, only few studies rely on established leadership theory, which makes it difficult to draw conclusions regarding what kinds of leadership managers should perform and under what circumstances. In industrial and organizational psychology, transformational leadership and contingent reward have been identified as effective leadership styles for facilitating change processes, and these styles map well onto the behaviors identified in implementation research. However, it has been questioned whether these general leadership styles are sufficient to foster specific results; it has therefore been suggested that the leadership should be specific to the domain of interest, e.g., implementation. To this end, an intervention specifically involving leadership, which we call implementation leadership, is developed and tested in this project. The aim of the intervention is to increase healthcare managers’ generic implementation leadership skills, which they can use for any implementation efforts in the future.

**Methods/design:**

The intervention is conducted in healthcare in Stockholm County, Sweden, where first- and second-line managers were invited to participate. Two intervention groups are included, including 52 managers. Intervention group 1 consists of individual managers, and group 2 of managers from one division. A control group of 39 managers is additionally included. The intervention consists of five half-day workshops aiming at increasing the managers’ implementation leadership, which is the primary outcome of this intervention. The intervention will be evaluated through a mixed-methods approach. A pre- and post-design applying questionnaires at three time points (pre-, directly after the intervention, and 6 months post-intervention) will be used, in addition to process evaluation questionnaires related to each workshop. In addition, interviews will be conducted over time to evaluate the intervention.

**Discussion:**

The proposed intervention represents a novel contribution to the implementation literature, being the first to focus on strengthening healthcare managers’ generic skills in implementation leadership.

## Background

There is a consensus that leadership is a key feature of implementation efforts. Leadership is included in most of the frameworks in implementation science that highlight factors essential to implementation, such as the Consolidated Framework for Implementation Research (CFIR) (cf. [1]) and the Preparation, Implementation, Sustainment (EPIS) framework [2]. These frameworks highlight that managers have an opportunity to influence several of the factors that are known to affect implementation success. From the existing implementation frameworks and from empirical findings on implementation leadership (particularly of evidence-based practice), it is clear that several manager activities and behaviors influence implementation [3–6]. This includes supporting employees, providing feedback, communicating about the implementation, influencing the work context, and serving as role models themselves [3–6].

Although it can be concluded that leadership is important for implementation, some shortcomings have been highlighted in the empirical studies on implementation leadership [3–6]. First, leadership has been defined and measured in different ways in the existing empirical studies, which makes comparisons across studies difficult. Second, few studies rely on established leadership theory; instead, most list specific activities managers should perform in relation to an implementation. The lack of theoretical underpinning for managerial activities makes it difficult to draw conclusions as to which activities managers should perform and why. Lists of managerial activities also neglect the fact that managerial activities are not performed in isolation from each other but are rather grouped in clusters of leadership behaviors, forming a more consistent leadership style. In addition, the need for studies on leadership styles was recognized in a recent call to develop concrete research evidence for key factors for implementation [7]. Taken together, based on the current research, it is difficult to draw uniform conclusions regarding what type of leadership is more effective for implementation and how to train managers in implementation leadership (cf. [4]).

### Full range leadership model

In research fields traditionally concerned with leadership studies, i.e., industrial and organizational psychology, the full range leadership model (FRLM) is the most comprehensive and most researched leadership model [8, 9]. The model aims to describe the full range of leadership behaviors, from the desired active leadership (called transformation leadership) to the undesired passive leadership (called laissez-faire leadership). Transformational leadership incorporates managers who act as role models and are able to formulate an inspiring vision for the future. They encourage employees to be creative and innovative, as well as giving them autonomy to make their own decisions, but at the same time coach them so that they are able to develop their abilities. Several meta-analyses and reviews have documented the positive effects of transformational leadership on productivity, employee effectiveness, job satisfaction, and group performance [10–15]. Studies in healthcare organizations have shown transformational leadership to be associated with better patient outcomes [16]. Moreover, transformational leadership has also been identified as particularly efficient for change processes [14, 17]. The full range leadership model also specifies transactional leadership, a rather active leadership behavior that is associated with mixed results due to the different natures of its sub-constructs. Of these, contingent reward has been found to be effective, whereas management by exception is generally not considered to reflect effective leadership behaviors. While transformational leadership seems valid to make change happen, it may not be sufficient. From research on organizational change, it is known that in addition to transformational leadership, the transactional leadership sub-category of contingent reward is important for the effective management of change processes, e.g., managers being specific, providing feedback, and evaluating the change process [17, 18]. These activities also align well with the leadership activities highlighted in the systematic review of leadership during implementation [3–6]. Moreover, the full range leadership model includes laissez-faire leadership—whereby managers do not take their leadership responsibility and instead act passively—which has been found to be related to negative outcomes [19]. To conclude, the combination of transformational leadership and contingent reward may be a valuable leadership style to test in the context of implementation leadership.

### Implementation leadership

Because the full range leadership model is a general leadership theory—that is, it describes how managers act in general rather than in relation to a specific aim—it has been questioned whether general leadership is sufficient for understanding the relation between leadership and specific results, e.g., implementation success. For example, managers who use transformational leadership do not necessarily focus on facilitating the implementation of a particular practice at their workplace but can instead focus their attention on other aims and still be perceived by their subordinates as transformational leaders. Therefore, leadership specific to the domain of interest has been suggested for securing the desired results in the specific domain [20]. This implies that scales measuring general transformational leadership, e.g., leadership behaviors without a specific target, might not be sufficient to capture the kind of leadership that facilitates implementation. In other research fields, domain-specific leadership has already been used to foster specific results at the workplace, e.g., to increase occupational safety [20] or health [21]. In the field of implementation, these types of studies are scarce, with the exception of Gifford et al. [22] or Aarons et al. [23, 24], who studied an implementation-specific form of leadership aimed at facilitating the implementation of evidence-based practice [23, 25]. Aarons used transformational and transactional leadership as the inspiration for his scale but did not keep the factor structure suggested by the full range model. Whereas Gifford aimed to change leadership, she assessed changes in leadership using interviews and also based the training on the CPE model of leadership [22, 26]. *To conclude*, for leadership to be relevant for implementation, managers need to focus their actions on the specific practice that is implemented, and hence need to show domain-specific leadership, which we call implementation leadership in the remainder of this study protocol. So far, only one scale for implementation leadership exists that strongly focuses on the implementation of EBP.

### Implementation leadership development

For organizations, it is not sufficient to know that implementation leadership is important; they also need to know how implementation leadership can be trained. Meta-analyses on the effects of leadership development have shown positive effects, e.g., that leadership behaviors can be enhanced through leadership training [14, 27]. More specifically, evaluations of interventions to foster general and specific transformation leadership have shown promising results, such as an increase in self- and employee-rated transformational leadership (for examples see [20, 28–32]). However, research on how to train managers in implementation leadership is scarce, an exception being a prior pilot study focusing on training managers in implementation leadership using evidence-based practice [23]. Aarons and colleagues found that the five managers who participated in the training showed a significantly greater change in behavioral routines, improvement in leadership behaviors, and an increased emphasis on evidence-based practice in interactions with their employees. Another prior study trained leaders in leadership related to the implementation of guidelines concerning diabetic home care. Managers in the experimental group showed a higher proportion of behavior change related to diabetic foot ulcers, which was the target of the practice implemented in this training. Moreover, through interviews, they could show that managers from the experimental group reported using more relation- and change-oriented behaviors. However, both these studies involved managers leading the implementation of specific practices (EBP) [23] and practice to prevent diabetic foot ulcers [22], rather than aiming to improve managers’ generic implementation leadership. In both trainings, the practice to be implemented (EBP or guidelines for diabetic foot ulcers) represented a large portion of the intervention’s content.

Whereas researchers are often interested in the implementation of a specific practice, managers are generally responsible for several implementations simultaneously [33]. Thus, increasing managers’ ability to lead implementations in general, rather than only in relation to a specific practice, is highly relevant from a practice perspective. In addition, from an organizational perspective, different managers are likely to be involved in different implementation efforts. It is not feasible to train managers in implementation leadership for each new practice to be implemented. Thus, it remains to be investigated whether and how generic implementation leadership can be trained with participating managers who focus on the implementation of different practices.

To summarize, based on psychological research, transformational leadership and contingent reward seem to be the most effective leadership styles during organizational change. There is also evidence that these styles can be trained, but there is little research focusing on training leaders in implementation leadership. Previous interventions of this kind [22, 23] have focused on one specific implementation initiative that all participating managers had in common. To our knowledge, no implementation leadership training has focused on generic implementation leadership. Therefore, an implementation object-independent training in implementation leadership has yet to be developed. This type of training is an important addition to the current trainings as it addresses the needs of organizations, which regularly conduct several implementations at the same time. Managers thus need to be able to lead the implementation of different practices. This goes over and above what existing trainings in implementation leadership have aimed for. In addition, exposing managers to different implementation objects within the same training structure emphasizes the general aspects of implementation leadership, supporting the development of effective implementation leadership that is less dependent on a specific implementation effort.

The aim of the present project is to develop and evaluate an intervention based on the full range leadership model for training managers’ implementation leadership. The intervention aims to improve managers’ generic implementation leadership by using their specific individual implementation case as a working example. This implies that the participating managers use different cases, based on what is relevant at their individual workplaces at the time of the training. Furthermore, to evaluate the potential effects of the intervention, a measure of generic implementation leadership based on the full range leadership model is developed. Experiential learning models are used to increase the transfer of these skills and behaviors from their specific case during the training to other implementation cases they will subsequently work on.

## Methods/design

### Setting

The study is carried out at the regional healthcare organization in Stockholm, Sweden. The Swedish healthcare system is tax-funded, and the provision of care is decentralized to autonomous regional authorities. The Stockholm regional healthcare organization encompasses care including primary, psychiatric, rehabilitation, and acute hospital care. The intervention is a collaboration between the researchers and the healthcare organization. The researcher’s role is to develop and evaluate the intervention, conducted by a knowledge center at the regional healthcare organization, the Unit for Implementation, where several of the research team members are employed. The intervention is funded with an independent, external research grant.

### Design

**Fig. 1:**
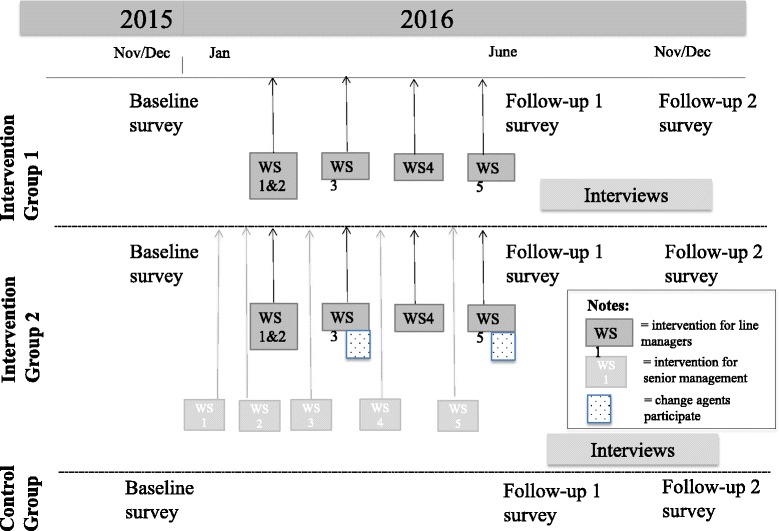
Timeline for the project

The study employs a non-randomized intervention design with two intervention groups and a control group. The intervention was tested with a pilot group before the actual intervention groups. The baseline survey was conducted in November/December 2015, and the intervention lasted from February 2016 to May 2016. The first follow-up surveys were conducted in June/July 2016, and the second in November/December 2016. In addition, continuous process evaluation with qualitative and quantitative data collection methods is conducted. For more details on the project timeline, see Fig. 1.

### Intervention groups

The two intervention groups are assigned based on the embeddedness in the organizational context. *Group 1* represents the typical intervention group used for most leadership trainings, consisting of managers who are interested in the topic and therefore volunteer to participate. Thus, group 1 consists of line managers from different divisions of the healthcare organization. All are currently implementing an evidence-based method or guideline, but the methods differ between the participants. *Group 2* consists of line managers from one division of the healthcare organization. The division’s senior management decided to allow all line managers to participate in the intervention, as a means to improve the implementation leadership of a newly established electronic system to facilitate care planning. Thus, the managers in this group are from the same division and implemented largely the same method. The c*ontrol group* consists of line managers from the same healthcare organization. They do not participate in an intervention, but responded to the questionnaires. The aim of including this group is to establish a reference value to indicate how much line managers develop their implementation leadership over time without input. The *pilot group* consists of 11 line managers who work as process leaders for development work within the primary care division. They receive the intervention a month ahead of the intervention groups, with the aim of testing the material and identifying possible need for modification.

### Recruitment process and participants

The target population is comprised of first- and second-line managers in the healthcare organization, e.g., the managers closest to the non-managerial staff or directly above the first-line managers. The recruitment of the participants in the two groups has been conducted as follows.

Group 1: An invitation to participate in the intervention was distributed using several communication channels, with the aim of reaching as many of the healthcare managers as possible. This included emails to the four division managers in the organization, emails to those on the Unit for Implementation’s mailing list (appx. 600 employees holding different positions) and a post on the Unit’s web site. The inclusion criteria were as follows: (1) holding a formal first- or second-line managerial position in the healthcare organization and (2) having a specific implementation project to actively work with during the intervention. The recruitment, conducted between June and October 2015, resulted in 41 potential participants. Three of these, who did not hold a formal managerial position, were excluded. Eleven applicants withdrew their participation due to major changes in their organization and a high workload. Moreover, four of the managers, who had contacted the research team asking to participate in the intervention, were later included in intervention group 2/the pilot group (see below). The final sample in group 1 consists of 21 managers.

Group 2: In 2015, the Unit for Implementation was approached by a division of the healthcare organization, requesting help with the implementation of a new electronic system to facilitate care planning. AR and HH met with the senior management group on five occasions during spring 2015 to discuss the possibilities for support from the Unit for Implementation. These meetings focused on clarifying and specifying the practice to be implemented and the organization’s needs and mapping where the organization was in their implementation process. It was determined that all 31 first-line managers would be offered a place in the implementation leadership intervention. Senior management was offered implementation leadership training similar to that of their line managers but adapted to the function of a senior management group. They agreed to participate, and this modified implementation leadership training was offered to all nine members of senior management for this division.

Control group: 50 managers, identified from a previously conducted questionnaire study by the Unit for Implementation as first-line managers, were sent an invitation to participate in the control group, e.g., the questionnaire. Of these, eight were excluded since they no longer worked as managers, and one was excluded from the control group since this manager had registered to participate in intervention group 1. Thus, 39 managers are included in the control group.

### Intervention development

The authors developed the intervention based on the scientific literature on leadership and leadership training and development as well as implementation. The scientific knowledge was collected through a systematic process. *The literature search* was conducted between January and May 2015 in PsychInfo, PubMed, Web of Science, and specific journals such as *Implementation Science*. The following search terms were used: leadership and implementation, leadership development, leadership training, and change management. This literature search gave us information regarding what type of leadership is regarded as the most effective, i.e., transformational leadership and contingent reward. In the implementation and change literature, we found strong support for the behavioral-focused approach to implementation, i.e., the framework of the Behavior Change Wheel [34]. Thus, the content of the intervention is based upon a combination of leadership theory (transformational leadership and contingent reward) and behavioral change theory (the Behavior Change Wheel) put in relation to the stages of implementation (from exploration to sustainment) [34, 35].

The scientific knowledge was completed with the views of *national experts in leadership and implementation* and *stakeholders in the local healthcare organization*, collected through the structured adaptive reflection methodology [36] in February and October 2015, respectively. Adaptive reflection, a technique used in higher education in order to create a common understanding of learning goals and activities [36], builds on the pedagogical theories of Kolb, Biggs, and Bloom [37–40]. The process starts with individual reflection over which skills, behaviors, and attitudes are required for a manager to lead implementation. This is documented on Post-it notes that the participants gather and then jointly sort in meaningful categories. Thereafter, the participants identify appropriate headings for each category of Post-it notes, using active verbs. In this way, the categories become the desired training outcomes. In addition, the line managers were asked to reflect on what kind of context they need within their division in order to successfully lead an intervention. This was also documented on Post-it notes and was used as the basis to form the content for the senior management intervention (described below). The experts were also asked to discuss relevant pedagogical models and methods for how the content of the intervention should best be taught to the managers. An adaptive reflection workshop was repeated for senior management in group 2, where they were given the opportunity to reflect on what they thought line managers in their division needed to be able to do in order to be a successful leader of the implementation. The results from the experts and the practitioners overlapped substantially.

### Intervention content

The intervention content is the same for groups 1 and 2. The content was pilot-tested with the pilot group, and only minor revisions were made. The intervention, consisting of five half-day workshops, aims at training managers in systematically implementing the practice they have chosen by applying the Behavior Change Wheel, an approach to achieving behavioral change while systematically applying relevant implementation leadership behaviors to facilitate the change process.

The central implementation leadership activities that are focused on are the following: identifying and defining the practice to be implemented, analyzing potential obstacles to the implementation, communicating the implementation, identifying relevant implementation leadership behaviors, handling resistance to the implementation, and evaluating and assuring the sustainability of the implementation. Each manager received personalized, 180-degree feedback containing information about general and implementation-specific leadership, contrasting the manager’s own rating with the aggregated employee ratings to allow them to identify their strengths and weaknesses in order to foster leadership development.

In summary, the intervention consists of the identification and analysis of what needs to be changed in terms of employee and managerial behaviors to enable successful implementation. A more detailed description of the content of each workshop can be found in Table 1.

**Table 1:** Content of the intervention (groups 1 and 2)

Workshops 1 and 2 Implementation and leadership 2 × 3 h	Workshop 3 Communicating the implementation 3 h	Workshop 4 Supporting the implementation 3 h	Workshop 5 Sustaining the implementation 3 h
Introduction to implementation and leadership (FRLM and Behavioral Change Wheel) Action plan initiation—identifying, pin-pointing and analyzing employee target behaviors 180-degree feedback on general and implementation-specific leadership behaviors, understanding and analyzing feedback on implementation leadership Introduction of assignment to work with between workshops 1/2 and 3	Follow-up on the between-workshop assignment Action plan finalization—identifying, pin-pointing and analyzing manager implementation leadership behaviors to enable and facilitate employee target behaviors Training of inspirational and motivational communication in relation to the action plans Introduction of assignment to work with between workshops 3 and 4	Follow-up on the between-workshop assignment Action plan follow-up and revision Understanding employee reactions and resistance to implementation Training of possible implementation leadership behaviors to overcome resistance and to support the implementation Introduction of assignment to work with between workshops 4 and 5	Follow-up on the between-workshop assignment Action plan follow-up and revision Intervention sustainment—measuring and monitoring change, conducting adaptations Implementation leadership to facilitate sustainment Planning for transfer of learning

### Workshops for senior management and change agents (only group 2)

The senior management intervention consists of five half-day workshops, aligned with the line manager intervention for group 2 (see Fig. 1). The overall theme of the workshops is the same as for line managers but adapted to the role of the senior management team. The basic idea was to train senior managers in behaviors concerning when and how to support their line managers in their implementation leadership. To create an alignment of the senior and line managers’ interventions, the senior management received a summary of the line managers’ Post-it notes corresponding to the topic of each workshop. Moreover, they received detailed information regarding what each workshop for line managers included and which exercises and between-workshop assignments they were doing. A more detailed description of the content of each workshop for senior management can be found in Table 2.

**Table 2:** Content of the senior management intervention

Workshop 1 Implementation and motivational inspiration 3 h	Workshop 2 Supporting the implementation 3 h	Workshop 3 Identifying obstacles and matching strategies 3 h	Workshop 4 Identifying obstacles and matching strategies 3 h	Workshop 5 Sustaining the implementation 3 h
Introduction to implementation and leadership (FRLM and Behavioral Change Wheel) Identifying and defining the implementation object Training of inspirational and motivational communication Introduction of assignment to work with between workshops 1 and 2	Follow-up on the between-workshop assignment Understanding employee reactions and resistance to implementation Training of possible implementation leadership behaviors to overcome resistance and to support the implementation Introduction of assignment to work with between workshops 2 and 3	Follow-up on the between-workshop assignment Analyzing the implementation target behavior through identifying hindering factors that may negatively affect the implementation Redefining the implementation object Introduction of assignment to work with between workshops 3 and 4	Follow-up on the between-workshop assignment Identifying, pin-pointing and analyzing senior management implementation leadership behaviors to enable and facilitate implementation in the whole organization Create an action plan Introduction of assignment to work with between workshops 4 and 5	Follow-up on the between-workshop assignment Action plan follow-up and revision Intervention sustainment—measuring and monitoring change, conducting adaptations Implementation leadership to facilitate sustainment Planning for transfer of learning

Change agents from each work unit, who have expert knowledge in the practice to be implemented in this division of the healthcare organization, were invited to participate during the line managers’ workshop 2, dealing with motivational and inspirational communication. They also attended a workshop of their own, dealing with implementation in general and leadership during implementation, where they could identify their role in the implementation process.

### Pedagogical approach

To promote a learning environment and to increase the transfer of training from the workshops to the workplace, the intervention was influenced by the theory of experiential learning [37] and by research on transfer of training [41]. The theory of experiential learning assumes mutual influence between theory and practice: concrete personal experiences are reflected upon, which advances the understanding of theoretical concepts relevant to personal experience. Further, an advanced understanding of certain theoretical concepts is translated into new actions, which leads to new personal experiences. These four steps constitute the learning cycle and have been incorporated in all workshops [37]. The pedagogical approaches used in each of the workshops are presented in Table 3. These approaches map onto Kolb’s steps. For instance, in the first two workshops, managers’ experience from their own implementation was combined with 180-degree feedback on implementation leadership; they reflected upon that experience; they received introductions to leadership and implementation theories through shorter lectures; and they experienced and tested new actions (either in practice during the workshop or through “cognitive experimentation” in terms of a discussion of lessons learned). Thus, the foundation throughout the intervention was constituted by the participants’ current implementation objects, which they continuously developed by applying the models or theories presented to them at each workshop. Moreover, they had the opportunity to practice desired leadership behaviors and receive feedback, which has been highlighted as important for the transfer of skills from training to practice [42, 43]. Consequently, implementation leadership was continuously practiced in role-play exercises, followed by constructive feedback from fellow participants and the workshop leaders. The role play also served as a demonstration for the observing participants, thus giving the participating managers the opportunity to learn from each other. In addition, interaction and learning among the managers was encouraged, for example by coaching each other, preparing for role play, and through reflection in small groups.

**Table 3:** Pedagogical approaches

Work with one’s own implementation object throughout the intervention
Short expert lectures
Reflection in small groups and individually
Group work
Role-play
Feedback from employees, i.e., 180-degree feedback
Feedback from fellow participants
Feedback from workshop leaders
Concrete work and help with one’s own implementation process, i.e., action plan and sustainability plan
Work at home between the workshops
Booster email between the workshops

### Data collection of process and effect measures

The effects of the intervention are evaluated through questionnaires distributed to the line managers and their employees at three time points in a pre-post design. A systematic process evaluation using a mixed-methods approach is conducted to capture the effects of the intervention, as well as to understand what works for whom and under which circumstances. The data sources for process evaluation include self-ratings in questionnaires, documentation from the intervention process, interviews, and a workshop evaluation in the questionnaire.

### Pre- and post-questionnaires to managers and employees

All line managers in the intervention groups and their employees receive an electronic survey sent to their work email addresses (pre = T1, post-intervention = T2 and post 6 months after the intervention = T3) (see Fig. 1). In the control group, managers but not employees receive the questionnaires. Before the baseline questionnaire was sent out, all the managers were sent an email to forward to their employees explaining the objective of the questionnaire and to encourage the employees to answer it. At baseline, all respondents received two reminders to participate in the survey during the time frame it was open. Subsequent to this, all the managers who had a low response rate (<70 %) at their unit were sent an additional reminder to encourage their employees to answer the questionnaire. The same procedure will be applied for the two post-measurements. All constructs included in the questionnaires are presented in Table 4.

**Table 4:** Constructs in the questionnaires

Construct	Data source	Number of items and response format (1 = strongly disagree to 5 = strongly agree if not *otherwise indicated*)	Respondent	Time of measurement
Primary outcomes				
Implementation-specific transformational, transactional and laissez-faire leadership based on [52, 53]	PPQ PEQ	20 items	M E	T1, T2, T3
Short form of implementation-specific transformational, transactional and laissez-faire leadership based on [52]	PEQ	6 items	M	WS: 1 and 2, 3, 4, 5
Changes in procedure based on [54]	PPQ	3 items	M E	T2, T3
Perceived change [55, 56]	PPQ	1 item 1 = large impairment–5 = large improvement	E	T2, T3
Secondary outcomes				
General transformational leadership and contingent reward transactional leadership based on [57, 58]	PPQ	9 items (2 items)	M E	T1, T2, T3
Implementation climate based on [59, 60]	PPQ	6 items	M E	T1, T2, T3
Quality improvement implementation scale based on [61]	PPQ	4 items	M E	T1, T2, T3
Self-rated health [62–64]	PPQ	1 item 1 = excellent–5 = poor	M E	T1, T2, T3
Vertical trust [62–64]	PPQ	4 items	M E	T1, T2, T3
Discomfort with work [65, 66]	PPQ	1 item 1 = never–5 = everyday	M E	T1, T2, T3
Work engagement [67, 68]	PPQ	3 items (1 item for each subscale)	M E	T1, T2, T3
Job satisfaction ([69], based on [70])	PPQ	3 items	M E	T1, T2, T3
Stress reaction and recovery [62, 64, 65]	PPQ	2 item 1 = never–5 = everyday	M E	T1, T2, T3
Self-rated productivity [71, 72]	PPQ	3 items 1 = low–10 = high	M E	T1, T2, T3
Group process [73]	PPQ	4 items	M E	T1, T2, T3
Job crafting based on [74, 75]	PPQ	8 items	M	T1, T2, T3
Qualitative job insecurity based on [76, 77]	PPQ	12 items	M E	T2, T3
Process evaluation				
Fit of the intervention [78]	PPQ	1 item	M E	T2, T3
Direction [78]	PPQ	2 items	E	T2, T3
Opportunity [78]	PPQ	1 item	E	T2, T3
Participation quality [53]	PPQ	3 items	M E	T2, T3
Integration into existing processes and structures [78]	PPQ	2 items	E	T2, T3
Readiness for change [53]	PEQ	5 items	M	WS: 1 and 2
Situational motivation [79]	PEQ	9 items	M	WS: 1 and 2
Knowledge developed by the authors	PEQ	6 items	M	WS 1 and 2, 3, 4, 5
Workshop evaluation developed by the authors	PEQ	14 items 1 = not valuable–10 = very valuable	M	WS 1 and 2, 3, 4, 5
Organizational support [80]	PEQ	4 items	M	WS 1 and 2, 3, 4, 5
Overall evaluation of workshop quality (Fricdrich A, Jenny GJ, Bauer GF: Development of a generic process appraisal scale for organizational health intervention elements, submitted)	PEQ	10 items	M	WS5
Overall outcome expectancy [81]	PEQ	3 items	M	WS5
Overall support of needs (Tafvelin S, Lundmark R, Stenling A: Development and validation of a measure of need supportive implementation scale, in preparation)	PEQ	8 items	M	WS5

*Note*: Data sources: PPQ = questionnaire, PEQ = workshop survey; Respondent: M = managers, E = employees; Measurement: T1 = baseline questionnaire, WS = workshop (during intervention), T2 = straight after intervention, T3 = 6 months after intervention

#### Main outcomes (primary and secondary outcomes)

The primary outcomes of the interventions are employee ratings of implementation leadership and changes in procedure. Secondary outcomes include employees’ and leaders’ work-related well-being and productivity, as well as implementation climate (see Table 4). Managers’ self-ratings of their general leadership are viewed as intermediate outcomes. A new measure of implementation leadership based on the full range leadership model was developed for the purpose of evaluating the outcomes of the intervention. Since the current training of managers had the goal of improving their generic skills in implementation-specific leadership, a measure specific to this aspect was needed. The previous measure for implementation leadership [24] focuses more on the implementation-specific leadership to implement evidence-based practice; hence, it is not possible to use it for measuring generic implementation leadership. Moreover, as this study is based on the full range leadership model, it has been important to keep the factor structure of our implementation leadership scale in line with the model. This is the other distinction from the implementation leadership scale developed by Aarons and his colleagues [24] and was the motivation to create a generic implementation leadership scale.

### Process evaluation questionnaires to participating managers

Short questionnaires were distributed before and after each workshop for the participating managers (see Table 4). In addition, the following constructs were included as potential mediators/moderators and were measured in the pre- and post-questionnaires to managers: frequency of manager-employee interaction, implementation experience, prior experience of leadership development, tenure at the workplace, tenure as manager, number of subordinates, gender, education, professional background, and age.

#### Interviews in process evaluation

The process evaluation also includes semi-structured interviews with participating managers and their employees. The main focus of the interviews will be on the transfer of training. Respondents are recruited using a purposeful sampling, so that different workplaces and individuals with different experiences of the intervention are represented. Ten to 20 line managers will be interviewed. The interviews will be conducted directly after the intervention and again approximately 6 months post-intervention. Moreover, interviews will be conducted with employees of line managers participating in the intervention (approximately 20 employees). The employees will be interviewed 3 to 5 months after the last workshop.

### Data analyses and power

Quantitative data (the pre- and post as well as the process evaluation questionnaires) are analyzed through descriptive analyses (e.g., frequencies, mean, and correlations), as well as with more complex analysis methods such as multi-level modeling and structural equation modeling. Multi-level modeling is used to account for the dependence of the data that is created by employees being nested within work units [44]. Structural equation modeling [45], e.g., confirmatory factor analysis, will be used to test the factor structure of the constructs included in the data collection. Moreover, SEM will be used when testing the relation of constructs with each other cross-sectionally as well as over time. The software packages used for these analyses are SPSS 23, HLM 7.1, and Mplus 7.2. In order to have enough power to conduct the relevant analyses, we follow the recommendations by Hox and his colleagues [46] regarding multi-level data, that 20 higher-order units—e.g., line managers—are sufficient to be able to analyze data with the relevant analysis techniques.

Qualitative data are audiotaped and transcribed verbatim. The data material is coded according to different evolving topics [47]. NVivo is used for the data analysis. Both types of data reveal different information that is important for understanding how well the intervention worked and which effects it had on line managers’ implementation efforts in their organizations. We therefore aim to connect the results of the two types of data in order to obtain a more holistic understanding and evaluation of the implementation leadership intervention.

### Ethical considerations

For the questionnaire data, all participants received a letter from the researchers explaining the purpose of the questionnaire. Moreover, a research plan outlining the overall project was included. On the first page of the online questionnaire, respondents received information on how to fill it out and were asked to give informed consent, confirming that they had understood the aim of the survey, that participation was voluntary and that they could withdraw their participation at any time, and that they consented to their response being used for research purposes. They were also ensured that no individual data would be reported or published. For the process evaluation and for the interviews, participants received all the abovementioned information and gave their written consent. Data collection within the project has been approved by the local ethics committee (ref no. 2015/857-31/5).

## Discussion

This project aims to contribute to implementation science by investigating implementation leadership, particularly the training of managers in implementation leadership. We combine and address two important topics in healthcare, i.e., implementation and leadership, and will thereby make several contributions to research and practice. First, the project will increase our understanding of how and under what conditions implementation leadership can be trained the most efficiently; e.g., it may be important to incorporate group processes and workplace factors when analyzing the broader conditions under which the training was successful. In the long run, understanding these mechanisms may increase the rate of successful implementation in healthcare. Second, we will gain a deeper understanding of which individuals participating in leadership training interventions is meaningful. The two intervention groups in this project differ in one aspect: do the managers participate alone (i.e., show interest in the training and applied for it) or do they participate together with other managers from the same organization (i.e., have varying interests in the training but organizational support)? Understanding how to best select and include managers in such an intervention will create valuable knowledge that can inform future interventions. Third, in this project, we used a technique from pedagogy, i.e., adaptive reflection, as a means to define training goals and activities in a systematic way. Involving practitioners in this way has several advantages: it is a way to validate the intervention and the learning goals that have been created based on scientific knowledge, and it creates engagement by the target group as they have participated in shaping the intervention and it is tailored to their needs. Fourth, we have included a variety of secondary outcome variables that relate to the productivity and well-being of employees and managers. This gives us the opportunity to investigate the effect of leadership training on outcomes related to employee well-being and productivity. Lately, leadership training has been discussed as an organizational intervention to increase employee well-being [48]. Particularly when training managers in transformational leadership, employee well-being is expected to be an important outcome of the intervention [49–51]. In comparison to other interventions, this project allows these kinds of effects to be evaluated. Fifth, the project contributes to the development of measures in implementation science. We have constructed, and will test, a new scale measuring implementation leadership. The scale is based on the full range leadership model, which encompasses transformational leadership, one of the most studied leadership styles in all investigations of leadership [8, 9]. It differs from the previous measure of implementation leadership [24] in that it measures generic skills in implementation-specific leadership and can hence be used to investigate leadership in relation to every new implementation. Whereas the previous measure of implementation leadership focused on implementation-specific leadership for one practice, namely EBP (evidence-based practice), it is not possible to use this measure for generic implementation leadership. Moreover, as research on leadership has identified effective leadership styles, e.g., transformational leadership and contingent reward, we have held it important that our newly developed measure closely follow the full range leadership model and its factors structure. This is the other distinction from the implementation leadership scale developed by Aarons and his colleagues [24], who was inspired by transformational leadership but then focused on leader behaviors that were important for the implementation of EBP, and in the scale does not follow the factor structure depicted in the full range leadership model. If the current scale proves to be a valid measure, it can be used in the future to measure implementation leadership for all kinds of implementation processes healthcare organizations are faced with. As feedback and monitoring are important for leadership development and behavioral change, it is important to have a valid measure that can be used both in practice—e.g., in training to provide feedback reports to help managers identify their strengths and weaknesses—and in research.

### Limitations

The recruitment processes for participating managers may have been limited; individuals could not be randomized into interventions and control group, as the project had aimed to do. Thus, we cannot exclude the possibility that the intervention groups systematically differ in some aspect. However, the longitudinal multi-source data give us the possibility to understand whether there are any third variables involved that might have caused differences between the groups and their development during the intervention.

The recruiting, whereby individuals either themselves applied to participate in the intervention (group 1) or were participating together with all their manager colleagues in the organization (group 2), might have resulted in great variations in motivation and readiness for the intervention between the groups. It might be the case that managers who themselves showed interest in participating in the intervention might have had a higher readiness for it and been more motivated to apply the new behaviors at work. However, potential differences can be detected through our process measure of motivation and readiness for the intervention.

## Abbreviation

FRLM, full range leadership model
